# Associations between Meal Patterns and Risk of Overweight/Obesity in Children and Adolescents in Western Countries: A Systematic Review of Longitudinal Studies and Randomised Controlled Trials

**DOI:** 10.3390/children11091100

**Published:** 2024-09-07

**Authors:** Georgios Saltaouras, Athanasia Kyrkili, Eirini Bathrellou, Michael Georgoulis, Mary Yannakoulia, Vasiliki Bountziouka, Urška Smrke, George Dimitrakopoulos, Meropi D. Kontogianni

**Affiliations:** 1 Department of Nutrition and Dietetics, School of Health Sciences and Education, Harokopio University of Athens, 17671 Athens, Greece; gsalt@hua.gr (G.S.); akirkili@hua.gr (A.K.); ebathrellou@hua.gr (E.B.); mihalis.georgoulis@gmail.com (M.G.); myianna@hua.gr (M.Y.); 2 Computer Simulation, Genomics and Data Analysis Laboratory, Department of Food Science and Nutrition, University of the Aegean, 81400 Lemnos, Greece; vboun@fns.aegean.gr; 3 Department of Cardiovascular Science, College of Life Science, University of Leicester, Leicester LE1 7RH, UK; 4 NIHR Leicester Biomedical Research Centre, Glenfield Hospital, Leicester LE3 9QP, UK; 5 Population, Policy and Practice Research and Teaching Department, GOS Institute of Child Health, University College London, London WC1N 1EH, UK; 6 Faculty of Electrical Engineering and Computer Science, University of Maribor, 2000 Maribor, Slovenia; urska.smrke@um.si; 7 Department of Informatics and Telematics, School of Digital Technology, Harokopio University of Athens, 17671 Athens, Greece; gdimitra@hua.gr

**Keywords:** childhood obesity, metabolically unhealthy obesity, meal patterns, breakfast, meal frequency, meal context

## Abstract

Childhood overweight/obesity (OV/OB) is a major public health problem in Western countries, often accompanied with comorbidities (e.g., hypertension and insulin resistance) (i.e., metabolically unhealthy obesity—MUO). Among diet-related risk factors of OV/OB risk and MUO, meal patterns remain limitedly studied. The aim of this systematic review was to explore associations between meal patterns and the risk of childhood OV/OB and MUO in children/adolescents aged 2–19 years. Longitudinal studies and randomised controlled trials from PUBMED and Scopus published between January 2013 and April 2024 were retrieved. Twenty-eight studies were included, all of which reported on OV/OB risk, with none on MUO risk. Regular consumption of breakfast (*n* = 3) and family meals (*n* = 4) and avoiding dining while watching TV (*n* = 4) may be protective factors against childhood OV/OB, whereas meal skipping (primarily breakfast; *n* = 4) may be a detrimental factor. Mixed effects of meal frequency on OV/OB risk were observed; no effects of frequency of lunch or of fast-food consumption and of meals served at school were found. There was insufficient evidence to support the role of other patterns (meal timing, eating in other social contexts). Meals were mainly participant-identified, leading to increased heterogeneity. Research focusing on childhood MUO and the use of harmonised definitions regarding the assessment of meal patterns are highly warranted.

## 1. Introduction

Childhood obesity remains one of the most significant global public health challenges of the 21st century. According to the Non-Communicable Diseases Risk Factor Collaboration, from 1990 to 2018, the age-standardised prevalence of childhood obesity increased in the vast majority of countries worldwide (93% of countries in girls and 98% in boys) [1]. The prevalence of overweight (including obesity) and obesity in Europe remains at alarmingly high rates (29% and 13% in boys aged 6–9 years; 27% and 9% in girls aged 6–9 years, respectively), with significant variations among countries [2], while in the U.S.A., a 20-year analysis highlighted a significant increase of about 30% in the prevalence of obesity and almost a 2-fold increase in severe obesity (defined as body mass index (BMI) ≥ 120th percentile of U.S. Centers for Disease Control and Prevention growth charts in the U.S.A.) in children aged 2–19 years [3].

Western countries (i.e., countries in Europe, North America, Oceania) share common exposures to childhood obesity such as early life exposures/perinatal factors, growth trajectories, socioeconomic factors, physical environment and lifestyle habits (diet, physical activity, sedentary activities, sleep) [4,5]. Regarding food intake, Western countries also share common food environments which are the “physical, economic, political and socio-cultural context in which consumers engage with the food system to make their decisions about acquiring, preparing and consuming food” [6]. Within modern Westernised food environments, traditional healthy dietary patterns, such as the Mediterranean diet, have been gradually abandoned, due to urbanisation, increasing affluence and the progressive globalisation of food supply, a phenomenon known as “nutrition transition” [7]. Therefore, the current Western lifestyle is characterised by the industrialisation of food and a lack of time for food preparation, which has led to increased consumption of ultra-processed foods and ready-to-eat meals [8] either within or outside the home.

Although the role of diet in childhood overweight/obesity (OV/OB) has been variously studied in terms of nutrient and food group intake or adherence to dietary patterns [9,10,11], there is an interest in other approaches to examine relationships between diet and health or disease that also capture other dimensions of eating habits, such as consumption of meals and snacks. A dietary approach in the context of meals, e.g., meal preparation, could complement dietary consultations and may be a more practical and easy way to convey clear messages to children and families. The shift in dietary/lifestyle patterns in Western and Westernised populations has also affected the frequency and quality of family meals, which in turn have been found to be important social settings for shaping children’s eating behaviours from a young age [12].

A “meal pattern” has been used in the literature as a term to describe an individual’s dietary habits either at the level of meals or eating occasions [13,14]. The term “eating occasion”, “eating event” or “eating episode” describes any consumption of food based on specific characteristics, such as the time of day, energy content, and combinations of foods, usually discerning eating occasions as main meals (i.e., breakfast, lunch, dinner) and snacks [13]. Meals have also been described in relation to their format (i.e., combination of foods or content of nutrients) and the context/environment the meals are consumed in (i.e., with the presence of other people or while performing an activity) [14]. There is, however, heterogeneity in the assessment of meal patterns in the literature, as different definitions of a meal have been applied across studies. A meal can be participant-defined through self-reporting [15] or it is defined according to the time it is consumed within the day [16], according to the energy content [17] or a combination of both [13], which may affect the interpretation of the findings [13].

In relation to childhood OV/OB, published systematic reviews have highlighted the role of different aspects of meal patterns in the risk of OV/OB, but evidence derives primarily from cross-sectional studies. For example, a meta-analysis of 75 studies (73 of which were in Western countries) exploring associations between frequency of family meals and children’s health showed that having frequent family meals was associated with a lower BMI and better overall diet quality; nevertheless, 89% of included studies had a cross-sectional design [12]. Similarly, an earlier meta-analysis of ten cross-sectional studies and one case–control study showed an inverse association of eating frequency (defined as the total number of eating episodes per day) and childhood OV/OB status in boys, but not girls [18]. The systematic review conducted by Monzani et al. [19] included 37 articles (27 in Western countries), 32 of which were cross-sectional, and it reported that skipping breakfast was associated with an increased risk or prevalence of OV/OB [19]. A meta-analysis that examined the association of meal timing and adiposity showed weak associations between higher energy intake close to bedtime and evening meal skipping with adiposity, but 17 out of 20 included studies had a cross-sectional design [20]. To date, there is no systematic review to capture different dimensions of meal patterns and their associations with the risk of developing childhood OV/OB in Western countries based on findings from longitudinal or randomised intervention studies that allow exploration of causality.

An emerging issue in relation to childhood OV/OB is the development of associated comorbidities, such as diabetes, hypertension, lipid abnormalities and liver dysfunction, which are often used to define metabolically unhealthy obesity (MUO) [5,21]. Children and adolescents with obesity have an increased prevalence ratio of 1.4 to develop prediabetes, 21.2 for cardiovascular disease and 26.1 for metabolic-associated steatotic liver disease, compared to children with normal weight [21]. Children with obesity are also more likely to live with obesity in adult life, which is associated with comorbidities across the life course [5]. The role of diet, let alone of meal patterns, in relation to MUO in children and adolescents has been scarcely explored, and very few interventions have evaluated markers of MUO [22,23,24].

Meal pattern consumption remains an interesting dimension of eating habits that could contribute to the engagement of optimal behaviours and lifestyle modification within the context of preventing and managing overweight and associated comorbidities. The aim, therefore, of this review was to systematically gather all available evidence from longitudinal cohorts or randomised interventions exploring effects of meal patterns on the risk of developing OV/OB and MUO in children and adolescents.

## 2. Materials and Methods

This systematic review was conducted according to the Preferred Reporting Items for Systematic reviews and Meta-Analyses (PRISMA) guidelines [25,26] (Supplementary Material S1: PRISMA 2020 Checklist). The study was registered in the PROSPERO international prospective register of systematic reviews of the National Institute for Health and Care Research (Title: The association of meal patterns and risk of obesity and metabolically unhealthy obesity in children and adolescents; registration number CRD42023477708).

### 2.1. Search Strategy

A systematic search was conducted in May 2024 in two databases (MEDLINE/Pubmed, Scopus) to identify studies which evaluated the role of meal patterns in childhood and adolescent OV/OB risk and MUO risk, published within the last decade (specifically January 2013–April 2024). Research questions and search keywords were guided from the “Population, Exposure, Comparator, Outcome” (PECO) model for epidemiological studies and the “Population, Intervention, Comparator, Outcome” (PICO) model for interventional studies (Table 1) [27]. Selection of exposure parameters was guided by Leech et al. [14] and included frequency of/omitting meals, consumption of meals within different contexts (e.g., while watching TV) and environments (e.g., at home) and meal quality. Outcome measures related to OV/OB included anthropometric indices, BMI/BMI z-score and body composition parameters. Indicators for MUO included blood pressure, blood lipids, glucose metabolism and metabolic comorbidities. Keywords were formulated according to the PECO/PICO model and are available in Supplementary Material S2. 

### 2.2. Eligibility Criteria

Inclusion criteria reflected the research questions, i.e., the population, the exposures/interventions and the outcomes of interest, as well as the study design. Original, peer-reviewed articles on children and adolescents (age range 2–19 years old as defined by the World Health Organisation [28] for baseline and follow-up), published from 2013 to 2024, that evaluated outcomes related to OV/OB or MUO were included. Only longitudinal studies and randomised controlled clinical trials (RCTs) were included to ensure a better quality of methodological design that could also allow aetiological assumptions. A minimum follow-up of 12 months was also applied to both study designs, which was deemed adequate to observe meaningful effects of meal patterns on OV/OB and MUO risk. The search was also refined to only include studies conducted in Western countries (i.e., Europe, U.S.A., Canada, Oceania), sharing common socioeconomic, physical and food environments.

Studies conducted on animals and people <2 or >19 years old; studies conducted in Asia, Africa and South America; reviews, letters, editorials, review protocols and pre-prints; cross-sectional studies; in vitro/in vivo animal or in silico studies; and non-randomised, uncontrolled clinical trials were excluded. Studies with multidisciplinary lifestyle observations or interventions with no clear analysis of the association between meal patterns and risk of OV/OB or MUO were also excluded. Due to the nature of the research question, studies evaluating meal patterns and MUO indicators in children without OV/OB at baseline and/or follow-up were also excluded.

### 2.3. Selection of Studies and Data Extraction

All studies identified from databases were imported in the Zotero software “https://www.zotero.org (accessed on 1 June 2024)”. Following the removal of duplicates, studies were screened for eligibility in two stages. Initially, a title and abstract screening was performed independently by two researchers (GS and AK). After exclusion based on titles and abstracts, all remaining articles were considered for full-text review by both researchers, who applied the eligibility criteria for the final selection. Disagreements at any stage were resolved by a third researcher (EB).

Data extraction was performed by one researcher (GS), with a second researcher (AK) randomly checking a sample of the eligible reports. Any disagreement was resolved by a third researcher (EB). Extracted information included the following:

– Study information: first author’s name, year of publication, acronym, country, setting, duration;
– Population: sample size, baseline age and sex distribution;
– Exposure(s) where relevant: type, definition, assessment method;
– Intervention (where relevant): groups, randomization, components, mode of delivery, duration;
– Outcome(s): type, definition, assessment method;
– Statistical analysis: analysed sample, statistical model, covariates;
– Study results: main findings.

Throughout the process, in case of missing information or uncertainties, relevant information was sought in Supplementary Materials or directly from study investigators. Extracted information is presented according to study design (prospective epidemiological studies or RCTs).

### 2.4. Risk of Bias

One researcher (GS) assessed the quality of all included studies and a second researcher (AK) assessed a random 20% of the sample. The quality assessment of prospective epidemiological studies was conducted with the Risk Of Bias In Non-randomized Studies—of Exposures (ROBINS-E) tool “https://www.riskofbias.info/welcome/robins-e-tool (accessed on 10 June 2024)” [29]. The ROBINS-E tool comprises seven domains: bias due to confounding; bias arising from measurement of the exposure; bias in the selection of participants in the study (or in the analysis); bias due to post-exposure interventions; bias due to missing data; bias arising from measurement of the outcome; and bias in the selection of the reported result. Each domain and the overall study are assessed as “low risk”, “some concerns”, “high risk” or “very high risk”.

The quality assessment of randomised controlled trials was conducted with the revised Cochrane risk-of-bias tool for randomised trials (RoB 2) tool “https://www.riskofbias.info/welcome/rob-2-0-tool/current-version-of-rob-2 (accessed on 10 June 2024)” [30]. The RoB2 tool comprises five domains: bias arising from the randomization process; bias due to deviations from the intended interventions (effect of assignment to intervention or adhering to intervention); bias due to missing outcome data; bias in the measurement of the outcome; and bias in the selection of the reported result. Each domain and the overall study are assessed as “low risk”, “some concerns” or “high risk”.

## 3. Results

**Figure 1:**
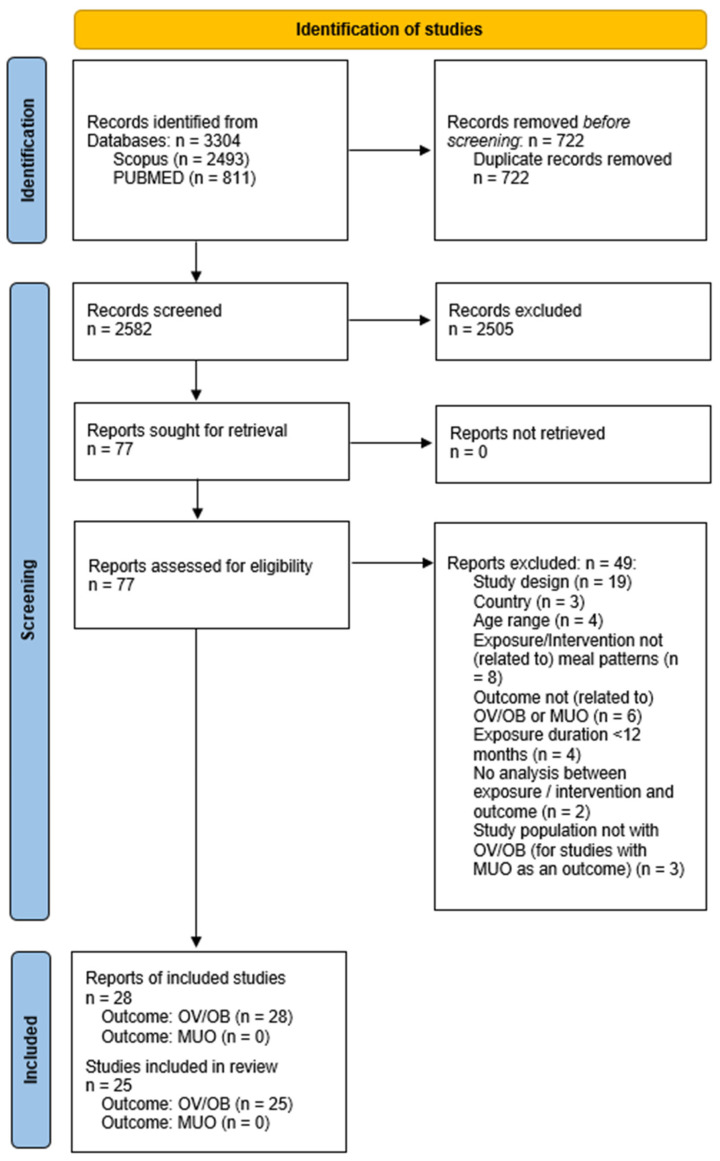
PRISMA flow diagram for meal patterns, overweight/obesity risk and metabolically unhealthy obesity risk. OV/OB: overweight/obesity; MUO: metabolically unhealthy obesity.

The initial search yielded 3304 results (2493 from Scopus and 811 from MEDLINE/Pubmed). After the removal of duplicates (722), 2505 articles were excluded following a review of titles and abstracts, and a further 49 were excluded after full-text examination. In total, 28 reports from 25 studies were included in this review (Figure 1). All reports examined associations between meal patterns and childhood OV/OB risk. No studies were found in relation to MUO risk.

### 3.1. Study Characteristics

Of the 28 included reports (25 studies), 13 studies were conducted in the U.S.A. [23,31,32,33,34,35,36,37,38,39,40,41,42], 12 in Europe (the U.K. [43,44,45], the Netherlands [46,47], Germany [48,49], the Republic of Ireland [50], Spain [51], Norway [52], multicentre across different European countries [53,54]), 1 in Australia [55] and 1 in New Zealand [56]). Finally, one study presented data from independent studies in different countries (Germany, the Netherlands, the U.K., the U.S.A.) [57]. All but one study had a prospective observational design, with a follow-up range between 1 and 10 years, and one study employed a RCT design [42], with a follow-up of 2.5 years. Analytic sample sizes ranged from 116 to 23,307 participants. The majority of studies recruited children and adolescents from school settings [32,33,34,35,36,37,38,40,41,42,47,48,49,50,51,52,54,55,57], and fewer from clinics [23,31,35,46,56] or the general population [39,43,44,45,57], while one study did not provide relevant information [53]. Most studies focused on school-aged children (*n* = 9) [32,33,34,40,41,48,49,50,54], while others included both pre-schoolers and school-aged children (*n* = 7) [23,31,38,43,44,53,57], both school-aged children and adolescents (*n* = 5) [39,42,47,51,52], only pre-schoolers (*n* = 4) [35,45,46,56] and only adolescents (*n* = 3) [36,37,55].

Regarding bias, most prospective observational reports had an overall high risk of bias, mainly due to risk of bias arising from measurement of the exposure (Table 2). For example, in several studies, data on exposures derived from self-reported questionnaires, often completed by parents/guardians. It was also unclear how meals were defined and whether clear instructions were provided to the participants on what constituted a meal. Only four reports raised “some concerns” in relation to bias [23,32,35,55] and no report had a low risk of bias. Most reports had a low risk of bias in relation to the selection of participants (28/28), post-exposure interventions (24/28) and the selection of reported results (23/28). The intervention included in this review [42] had a low risk of bias in four domains (randomisation process, missing data, measurement of the outcome and selection of reported result) and concerns raised in relation to one domain (deviation from intended intervention) (Table 2). 

### 3.2. Main Exposures

A number of different exposures in relation to meal patterns were identified. The most commonly studied exposure was frequency of consumption of/skipping specific meals, such as breakfast [23,32,34,36,37,39,40,44,46,47,48,49,50,54,57], dinner/evening family meals [23,32,37,46,54], and lunch at home or at school [32,33,39,46,54]. In most studies, meals were self-reported without evidence of a clear, objective definition on the timing or the content of a meal consumed. One study provided a definition of breakfast [34], that is, “the first meal in the morning consisting of any solid food, beverages, or both and named by the respondent as “breakfast”. In one study [37], participants were asked how often they had breakfast “which was more than a glass of milk or fruit juice”, whereas in another study [36], they were asked what they usually have for breakfast “on a weekday morning”. Regarding lunch, two studies [32,33] defined school-provided lunch as “a full meal including salad, soup, a sandwich”. Relevant information about how meals were defined and assessed are included in Table 3 and Table 4.

Included studies also examined the association of the consumption of other meal patterns with childhood OV/OB, such as snacking [39,50] (one study [50] provided examples of snacks), eating fast foods [23,37,39,50,55] (three studies provided examples [37,50,55] of fast foods) and having sugary drinks in between meals [43]. Other exposures related to meal patterns included frequency of family meals [31,39], family meal interpersonal quality [31], regular timing of meals [43], eating meals while watching TV [23,37,39], eating while doing homework [39], eating alone [39], eating with friends [39], first and last eating events [35], mealtime setting [39,45] and patterns of breakfast location (combination of the variables “frequency” and “setting” in relation to breakfast) [41]. Some studies examined the association of meal frequency/eating occasion in childhood obesity; however, the definition of meal/eating occasion varied significantly among studies. In the study by Jaeger et al. [53], an eating occasion was defined as any occasion where food or beverages are consumed. In Taylor et al.’s study [56], a separate eating occasion was defined as “the start of the next meal or snack that had to be more than 15 min after the end of the previous meal or snack (i.e., separated by at least four five-minute blocks)”. Stea et al. [52] defined regular breakfast/lunch/dinner/evening meal consumers if eating all meals every day. Two studies assessed meal frequency as the combined frequency of breakfast and evening meals [33,38]. In one study, the frequency of eating meals with family was evaluated on a continuous scale from 0 (never) to 8 (>7 meals per week); however, it was unclear how family meals were defined [31].

Data on exposure(s) derived primarily from self-reported questionnaires completed by either parents/caregivers [23,32,33,44,45,46,48,49,50,52,54,57] or children/adolescents [23,36,37,41,47,51,55]. Two studies used dietary recalls [34,35], three studies used dietary records/diaries [39,53,56] and three studies collected data via interviews with parents/caregivers [38,40,43]. All but one study analysed the exposure of interest as a categorical variable; Jaeger et al. [53] explored meal patterns as the amount of energy intake (kcal) in predefined time slots. 

The only cluster RCT included in this review assessed the effect of eating breakfast in the classroom and of providing breakfast-specific nutrition education in comparison to having breakfast in the school cafeteria, on overweight and obesity among urban children in low-income communities. Data on exposure were collected by teachers (intervention arm) and cafeteria staff (control arm) [42].

### 3.3. Main Findings

Findings are presented in three axes, according to the definition of meal constructs as described by Leech et al. [14]:

(a) **Meal patterning**, including frequency of eating occasions, regularity of meals, meal skipping, meal timing, and spacing of eating occasions;
(b) **Meal format**, referring to food type, food combinations, or food sequencing;
(c) **Meal context**, related to the presence of others at a meal, eating while performing activities, meal location.

#### 3.3.1. Meal Patterning

Meal patterning essentially refers to the frequency or timing of eating occasions, either examining the number or the distribution of meals/snacks within the day or focusing on the regularity/skipping of a specific eating occasion, with special interest in breakfast. In relation to meal frequency, with different definitions used to describe the term, most studies showed no association with adiposity parameters. One study found that higher meal frequency (5 meals per day compared to 3 or fewer meals per day) at baseline was associated with a smaller increase in BMI-z score, a smaller increase in waist-to-height ratio and lower odds of developing obesity at follow-up [51]. However, no association of meal frequency with obesity risk [32] or change in BMI-z score at follow-up [38,52,56] was found in other studies. A dose–response association was observed between the regularity of mealtimes at age 3 and the risk of developing obesity at age 11 [43]. Specifically, compared to children who always had regular mealtimes, those who usually had regular mealtimes experienced a 23% reduction in the odds of obesity, while those who rarely or never had regular mealtimes had a 38% reduction [43]. Moreover, timing of the first eating episode at baseline was not associated with fat mass, fat-free mass or body fat% in children 3–5 years old at follow-up one year later [35]; however, the same study showed later timing of the last meal of the day at baseline to be associated with increased fat mass and body fat% at follow-up one year later [35].

Breakfast constitutes the most studied meal within meal patterning. Daily consumption of breakfast at baseline compared to less frequent consumption (<7 times/week) had a favourable association with BMI [23] and body fat mass % [23,46] at follow-up, whereas no association with odds of OV/OB [46] was observed. In de la Rie et al.’s study [57], such benefit (association with lower BMI) was evident in two of the four cohorts examined. Other studies showed no association of breakfast frequency with OV/OB incidence or/and prevalence [47,50] or change in BMI-z score [37,50]. Moreover, breakfast eating habits have also been assessed as skipping breakfast, with mixed results in relation to obesity risk, and a varying definition of the term across studies. One study used a yes/no variable [44], two studies assessed meal skipping as consuming it “never/rarely” compared to “often/always” [48,49], one study included the category “frequent skippers” as having a meal 0–3 times/week [41], another named a stable breakfast skipping pattern as “eating breakfast <7 times/week” consistently over time [46], and one study assessed meal skipping if participants reported they “did not eat” that meal (option 0) on a 0–7 times/week scale [36]. In most studies, skipping breakfast was associated with weight gain [44,48], increased waist-to-height ratio [48], increased BMI percentile [48], abdominal obesity [48], increased % of body fat mass [46], and increased overweight risk [48] and OV/OB risk [41]. However, skipping breakfast (yes vs. no) was also associated with decreasing weight trajectory, which was defined as a change in BMI category over time, i.e., from overweight at baseline to normal weight at follow-up [44]. In three studies, no association of skipping breakfast with overweight risk (in males) [36], obesity risk [36,48] or abdominal obesity risk [49] was observed. 

A child’s sex may also play a role, with some findings indicating a beneficial effect of breakfast consumption especially in girls. In a study with both sexes, breakfast consumption was associated with waist circumference, trunk fat mass and trunk to peripheral fat mass ratio only in girls [23]. Another study conducted only in girls, following them from childhood through adolescence, identified eating breakfast at age 9 (without specifying frequency) as a significant protective predictor against adiposity at age 19 [39]. Skipping breakfast (no breakfast consumption in any day of the week) was also associated with increased OV/OB risk only in girls in one study, but not in boys [36].

Consumption of lunch (as a yes/no variable) and frequency of its consumption were not associated with any parameter of adiposity [33,46,54], but skipping lunch at 9 years of age was identified as a significant risk predictor of adiposity in females at age 19 in one study [39]. Regarding dinner, eating dinner < 6 times/week was not associated with changes in fat mass or BMI over time compared to eating dinner every day [46].

#### 3.3.2. Meal Format

The energy and macronutrient content and consumption of specific foods have been examined within the meal format, with a special focus on snacks and fast-food consumption. One study investigated the time-of-day energy intake in relation to obesity risk and found that energy and macronutrient intake distributed in different eating occasions throughout the day was not significantly associated with BMI-z score [53]. Regarding the content of breakfast, higher-frequency consumption of ready-to-eat-cereals (RTECs, 3 vs. 1 times per week of breakfast RTEC) in fourth grade was associated with a decrease of about 2 percentiles in children’s BMI in sixth grade [34].

Children who abstained from sweet and savoury snacks at baseline had decreased OV/OB prevalence, decreased OV/OB risk and decreased change in BMI-z score at follow-up, compared to regular eaters on a daily basis [50]. On the contrary, consumption of sugary drinks in between meals was not associated with obesity risk in one study [44]. Similarly, consumption of fast foods/takeaway foods was not associated with changes in BMI [37,50,55], body fat % [55], waist circumference [55], odds of developing obesity [55] or change in OV/OB prevalence [50]. However, one study showed that consumption of fast foods less than once a week at baseline was associated with lower BMI-z score, waist circumference, body fat %, trunk fat mass and trunk to peripheral fat mass ratio at follow-up, but only in girls [23].

#### 3.3.3. Meal Context

The aspects of meal context studied were ‘eating with whom’, ‘what doing in parallel’, and ‘place of eating’ and, in particular, eating with family, eating while performing another activity, esp. screen use, and eating at school. Studies have mainly focused on breakfast and dinner, with scarce reports on lunch. 

Family meals were associated in one study with reduced obesity prevalence at follow-up, with each additional family meal significantly reducing obesity prevalence by 4% [31]. Two studies assessed eating breakfast with family, and both showed a beneficial effect [32,54]. Children who consumed family breakfast three to seven times a week at baseline had decreased odds of OV/OB two years later, compared to those who never had breakfast with family [54], while children with overweight at baseline and healthy weight at follow-up were more likely to eat breakfast together with at least one member of the family than children with overweight, both at baseline and follow-up [32]. Four studies examined the role of family dinner in obesity risk [23,32,37,54], two of which found a protective role, but the other two had no association. In one study, children who had family dinners three to seven times per week at baseline were more likely to have lower BMI and reduced odds of overweight/obesity two years later [54]. Similarly, daily family dinners, in comparison to non-daily dinners (≤6 times/week), were associated with decreased BMI-z score, waist circumference, trunk fat mass and trunk to peripheral fat mass ratio, in girls but not boys [23]. On the other hand, two studies found no association of frequency of family dinners and BMI over time [32,37].

Four studies examined the effect of watching TV while eating meals [23,37,39,45]. In particular, one study showed lower BMI-z score, waist circumference, body fat%, trunk fat mass and trunk to peripheral fat mass ratio in boys and lower body fat% in girls that ate meals while watching TV less than once a week, compared to those who ate meals in front of TV more frequently [23]. Greater mealtime screen use and less use of a table while eating a meal (described as intermediate and informal meal settings) were associated with increasing overweight weight trajectories (i.e., being at the “normal weight” category at baseline and at the “overweight” category at follow up), compared to lower mealtime screen use and eating a meal on the table (formal meal setting) [45]. One study, however, found that eating while watching TV at age 9 was a significant protective predictor of adiposity in females at age 19 [39], which was highlighted as “surprising” by authors, while no associations were observed in another study [37]. Also, a more positive interpersonal quality of family meals at baseline, characterised by the involvement of conversations without media distractions, was not associated with obesity prevalence at follow up, compared to a less positive interpersonal quality of family meals that involved more media distractions and less conversations [31]. Finally, in one study, eating with friends, eating while doing homework and eating in the bedroom were also identified as protective factors against adiposity in females, findings which have also been highlighted as “surprising” by the authors, who nevertheless commented that the findings may imply a mitigation effect of such eating behaviours when healthy snacks are offered, an assumption which might be supported by their finding of “parents fixing a snack” being a protective adiposity predictor [39].

Meals served at school have also been studied in regard to obesity risk [32,40,42]. Consumption of breakfast at school (yes/no) in fifth grade was not associated with obesity risk in eighth grade [40]; an intervention to increase breakfast consumption at school also had no effect in the combined OV/OB incidence or prevalence [42]. However, when the incidence and prevalence of obesity were examined separately, the incidence of obesity alone was higher in intervention schools than in control schools (11.6% vs. 4.4%; OR 2.43; 95%CI, 1.47–4.00), as well as the prevalence of obesity (28.0% vs. 21.2%; OR 1.46; 95%CI, 1.11–1.92) after 2.5 years of intervention [42]. Only one study examined the consumption of school-provided lunch (yes/no), and found that it was associated with decreased odds of weight gain at follow-up 3 years later [32]. In all studies, no information on whether a school-provided meal reduced meal skipping, replaced a meal eaten at home or was consumed in addition to a home-provided meal was mentioned

## 4. Discussion

Western countries, which share common socioeconomic, physical and food environments resulting in similar food availability and lifestyle habits, face increased risks for childhood OV/OB and related chronic non-communicable diseases [6]. Based on this perspective, the shift from traditional dietary patterns to Westernised dietary habits has resulted in changes in both food choices and patterns of food consumption, both of which have been suggested as potential OV/OB risk factors for children and adolescents [8]. In this systematic review, evidence on the associations of meal patterns with childhood OV/OB and MUO risk was collected. In the absence of published studies on MUO risk, only evidence on OV/OB was presented. To our knowledge, this is the first systematic review that captures different dimensions of meal patterns (patterning, format, context) and focuses on studies with a longitudinal design (prospective studies and randomised controlled trials), aiming to explore aetiologic associations between meal patterns and OV/OB and MUO risk. Regular consumption of breakfast and family meals, as well as avoiding watching TV while eating, may be protective factors against childhood OV/OB, whereas meal skipping (primarily breakfast) may be a detrimental factor. Mixed effects of meal frequency on OV/OB risk were observed. No effects were observed regarding frequency of lunch consumption or of fast-food consumption and of meals served at school, while there was insufficient evidence to support the role of other meal patterns such as meal timing and eating in social contexts other than family in OV/OB risk.

Despite methodological considerations, frequent/daily consumption of breakfast (or avoiding breakfast skipping) has been, according to current findings, highlighted as a protective factor against childhood OV/OB longitudinally, indicating a potential aetiologic association between breakfast consumption and OV/OB risk. Recently published systematic reviews of both cross-sectional and longitudinal studies are in agreement with these findings, particularly regarding breakfast skipping [19,58]. This constitutes an important public health message for preventive policies and practises, especially taking into consideration that the literature shows a discernible decline in breakfast consumption in children while entering adolescence [59]. Promotion of frequent/daily consumption of breakfast throughout childhood and adolescence would be an efficient strategy towards reducing OV/OB risk.

The self-reported data collection methods employed in most included studies might have variously impacted the current findings. For example, most of the studies did not provide guidance as to what constitutes a meal and a small number of studies used varied definitions, leading to ambiguities regarding the studies’ comparison and result interpretation. Participant-identified eating occasions are prone to subjectivity, as the interpretation and allocation of each eating event may vary significantly [14]. Future studies should ensure the use of robust definitions for meals, such as the proposed definition for breakfast by O’Neil et al. [60] as “the first meal of the day that breaks the fast after the longest period of sleep and is consumed within 2 to 3 h of waking; it is comprised of food or beverage from at least one food group, and may be consumed at any location”. The lack of a universally accepted definition of breakfast may prove challenging in the identification of pathways through which breakfast consumption may play a significant role in the development of childhood OV/OB and MUO. Also, the use of methodologically more reliable assessment tools for the evaluation of meals in future research, such as dietary records and recalls, will not only provide more reliable results on the role of breakfast/meal frequency, but also on the role of meal format/composition in the development of OV/OB.

Results from this review also highlighted a potential protective effect of family-shared meals against OV/OB risk, which is supported by older systematic reviews/meta-analyses of both cross-sectional and longitudinal studies that were conducted primarily in Western countries [12,61]. The parental influence in shaping children’s eating behaviours has been well documented, from either the perspective of parenting feeding practises, dietary habits or weight status [62,63,64]. Family-shared meals could be considered a factor under the construct of home organisation within the family environment, and associations have been found between family meal routines and childhood and adolescence obesity markers, after correcting for the moderating role of socioeconomic factors [65]. It would be interesting to explore which parameters of the family meal routines and family environment mediate this protective effect towards OV/OB risk. For example, an earlier cross-sectional study by Skafida et al. [66] showed that parameters such as children eating the same food as their parents, having conversations with parents during meals and having “enjoyable” mealtimes were positively associated with children’s diet quality, while eating with parents at the same time was not a significant predictor [66]. Only one study in this review [31] assessed interpersonal quality of family meals and specifically conversations with family during meals, compared to media distraction, with no significant results in relation to OV/OB risk. Future research should focus on the longitudinal effect of family meal routines and related parameters, which could also include food availability, food quality and consumption of homemade foods on the OV/OB risk. The family environment should also be considered as a potential target for future interventions that will focus on the improvement of the frequency and quality of family meals and explore their effect on weight status. 

Included studies that investigated the effect of eating meals while being distracted by media and particularly by watching TV on OV/OB risk indicated a protective effect when abstaining from TV while eating. Findings from a systematic review showed a positive association between TV viewing and consumption of energy-dense foods, such as pizza, fried food, sweets and sugar-sweetened beverages, and a negative association with consumption of fruits and vegetables [67]. TV viewing has been hypothesised to increase OV/OB risk through increased sedentary time [68,69], influence of TV advertisements of energy-dense foods [70], promotion of mindless eating during viewing [71], and increased snacking [69].

Although this review identified several longitudinal studies involving the family environment, considerably fewer assessed meal patterns in the school environment (in the form of school-provided meals); thus, there is insufficient evidence to support a positive or detrimental effect on children’s weight status. Evidence from two large free school meal programmes in the U.S.A., the Community Eligibility Provision and the Healthy, Hunger-Free Kids Act, have shown mixed results on childhood obesity trends [72,73]. Provision of free breakfast and lunch to students had led to a modest decrease in obesity prevalence in the Community Eligibility Provision programme within 5 years of implementation [72], whereas no effect was recorded in the Healthy, Hunger-Free Kids Act study on obesity risk [73]. These results may be due to the fact that it is unknown how school meals affect children’s daily meal and dietary intake, that is, whether they replace existing meals or are added to a given meal pattern, leading, probably, to increased dietary intake. Even though it may be unclear whether provision of meals at schools is associated OV/OB risk, such school programmes have documented improvements in diet quality [74], food security [75] and academic performance [75]. It would be essential for future research studying the effect of school-provided meals on childhood OV/OB risk to assess the quality and quantity of school-provided meals, including snacks, and explore whether they replace or are consumed additionally to the rest of the meals by also correcting for total energy intake as a confounding factor.

Some evidence suggests that sex might have a mediating role in meal patterns and OV/OB risk, and this was mainly observed in the literature exploring the effect of breakfast consumption. According to findings from two studies [23,36], regular breakfast consumption was associated with decreased adiposity markers, while skipping breakfast was associated with increased OV/OB risk in girls, but not boys. One of these studies also found a protective effect of daily family dinners on obesity markers in girls but not in boys. Mahmood et al. [54] suggested that a potential explanation for sex differences might be due to differences in dietary and social behaviours. Girls tend to eat more frequently with family and friends than boys [76], whereas boys eat more takeaway meals at home, compared to girls [77]. Also, girls may be more prone to societal influences of dieting, leading to increased prevalence of meal (particularly breakfast) skipping [76]. It is unknown whether dieting and social behaviours would explain sex difference in the included studies. One of the studies in this review explored the impact of dieting on OV/OB risk and found no associations regardless of sex, but the variable “dieting” was not included as a confounder for the association of breakfast skipping and OV/OB risk. The other study did not explore other dietary or social behaviours that could potentially explain the different results according to sex. The biological dimension of sex mediating the role between frequency of consumption of meals and OV/OB risk is also largely unknown and constitutes a potential area for future research.

It should also be noted that the current review identified only one RCT regarding meal patterns (provision of breakfast) and OV/OB markers and no studies for MUO risk. Given the importance of preventative measures against obesity-related comorbidities, future research should focus on the role of meal patterns and the family environment in the development of comorbidities in children with OV/OB. Also, future interventions could target aspects of meal patterning, format and/or context in preventive interventions.

This review has strengths and limitations. In order to promote reproducibility and transparency, the PRISMA guidelines and a detailed search strategy were implemented. The inclusion of most recent studies with a longitudinal design is considered a strength of the review, so evidence on potential aetiologic associations of the role of meal patterns and OV/OB risk is synthesised. The findings are, however, affected by the scientific quality of the included studies, presenting, in most of the cases, with high or very high risk of bias due to the large heterogeneity in the assessment of exposures [particularly deriving from self (parent)-reported questionnaires]. Also, the search was limited to two, widely available databases (Pubmed/MEDLINE and Scopus) and reference lists of included studies; it was not possible to conduct the search in databases where subscription was required. Finally, included studies were conducted in Westernised countries, limiting the generalisability of the findings in non-Westernised cultural settings.

## 5. Conclusions

Some evidence supports a protective role of regular/daily breakfast consumption, regular shared family meals and avoiding watching TV while dining against OV/OB risk, deriving mainly from longitudinal studies, while no relevant published study reporting on childhood MUO risk was identified. There was insufficient evidence of the role of other meals such as lunch, other meal patterns such as meal timing, and other social environments such as consuming meals at school, in OV/OB risk. Overall, the quality of the findings is poor due to the high bias of the included studies. The use of harmonised definitions for the assessment of different meals, as well as better methodological approaches, is warranted to provide more robust results in future studies. Future interventions should also target the family environment with a view of determining the protective parameters of shared family meals in OV/OB risk.

## Figures and Tables

**Table 1:** Population, Intervention/Exposure, Comparator, Outcome criteria.

Variable	Definition
Population	Children/adolescents: 2–19 years old
Exposure/ Intervention	High/low eating/meal and snack frequency; early/late timing of breakfast, lunch or dinner; low/high levels of omitting a meal; types of food eaten in different social contexts (alone vs. with others); types of food consumed while watching TV vs. other activities; meals consumed at home vs. out of the home; high/low meal composition/quality. Interventions promoting increased meal frequency; early timing; low levels of omitting meals; eating food with others; food consumed without activities; meals consumed at home; eating high-quality meals.
Comparator	Low/high eating/meal and snack frequency; late/early timing of breakfast, lunch or dinner; high/low levels of omitting a meal; types of food eaten in different social contexts (alone vs. with others); types of food consumed while watching TV vs. other activities; meals consumed at home vs. out of the home; low/high meal composition/quality. No intervention; intervention of a different meal pattern; standard care.
Outcome	OV/OB risk MUO risk

**Table 2:** Risk of bias assessment for the longitudinal studies and randomised controlled trial reviewed regarding the meal pattern factors associated with overweight/obesity risk.

**Study (Author, Year)**	**Risk of Bias for Longitudinal Studies**							
	**D1**	**D2**	**D3**	**D4**	**D5**	**D6**	**D7**	**Overall**
Anderson, 2017 [43]	−	++	−	−	−	−	+	++
Bel-Serrat, 2018 [50]	+	++	−	−	++	−	−	++
Berge 2023 [31]	−	+++	−	−	+	+	−	+++
Chang and Gable, 2013 [32]	+	+	−	−	−	−	−	+
Chang and Halgunseth, 2015 [33]	+	+++	−	−	+	−	−	+++
de la Rie, 2023 [57]	−	+	−	−	++	−	+	++
Balvin Frantzen, 2013 [34]	++	−	−	−	++	−	−	++
Gingras, 2018 [23]	+	+	−	−	−	−	−	+
Goetz, 2022 [35]	+	−	−	−	−	−	−	+
Gopinath, 2016 [55]	−	+	−	−	−	−	−	+
Jaeger, 2022 [53]	++	++	−	−	+	−	−	++
Juton, 2023 [51]	+	−	−	+	++	+	−	++
Kelly, 2016 [44]	++	+	−	−	+	+	+	++
Kesztyüs, 2016 [49]	+	++	−	+	++	++	−	++
Liechty and Lee, 2015 [36]	+	−	−	−	+	++	−	++
Lipsky, 2015 [37]	−	−	−	−	+	++	−	++
Loren, 2022 [38]	+	+++	−	−	−	−	−	+++
Mahmood, 2023 [54]	+	+	−	+	++	++	−	++
Narla and Rehkopf, 2018 [39]	+	++	−	−	−	+	++	++
Parkes, 2020 [45]	−	+	−	−	+	++	−	++
Stea, 2014 [52]	−	−	−	−	+++	+	−	+++
Sudharsanan, 2016 [40]	−	++	−	−	++	++	−	++
Taylor, 2017 [56]	+	++	−	−	+	−	−	++
Traub, 2018 [48]	+	++	−	+	+	++	−	++
Wang, 2017 [41]	+	++	−	−	+	−	−	++
Wijtzes, 2016 [46]	−	++	−	−	+	+	++	++
Winter, 2016 [47]	++	+++	−	−	+	+	−	+++
Di = domain of risk of bias, i = 1–7 as follows: D1: due to confounding; D2: arising from measurement of the exposure; D3: in selection of participants into the study (or into the analysis); D4: due to post-exposure interventions; D5: due to missing data; D6: arising from measurement of the outcome; D7: in selection of the reported result.								
**Study (Author, Year)**	**Risk of Bias for Randomised Controlled Trial**							
	**D1**	**D2**	**D3**	**D4**	**D5**	**Overall**		
Polonsky, 2019 [42]	−	+	−	−	−	+		
Di = domain of risk of bias, i = 1–5 as follows: D1: arising from the randomization process; D2: due to deviations from intended interventions; D3: due to missing data; D4: in measurement of the outcome; D5: in selection of the reported result.								

Symbol coding of risk of bias: − low risk of bias; + some concerns; ++ high risk; +++ very high risk.

**Table 3:** Characteristics of prospective/longitudinal epidemiological studies exploring the association between meal patterns and the risk of overweight/obesity.

Study	Population			Exposures	Outcomes	Covariates	Results
Author, Year, Country	N	Age ^a^	Sex ^b^				
Anderson, 2017, UK [43]	10,995	36.8 mo (36.3–37.7)	M: 5557 (50.3%) F: 5438 (49.7%)	Regular timing of meals (“Always” was coded as having a regular mealtime routine; “Never or almost never”, “Sometimes” and “Usually” were coded as inconsistent mealtime routine)	FU BMI z-score (IOTF) FU obesity (BMI z-score at or above 98.9th centile)	Birth weight, household income, household size, parental age at the time of child’s birth, ethnicity, parental academic and vocational qualifications, country (England, Wales, Scotland, Northern Ireland), bedtime routine, TV/video time routine, self-regulation	Decreased odds for developing obesity at age 11 when usually having meals at regular times compared to always having regular meals [OR (95%CI): 0.77 (0.62–0.97)]; when sometimes, almost never or never have regular meals vs. always having regular meals [OR (95%CI): 0.62 (0.41–0.94)]
Bel-Serrat, 2018, Republic of Ireland [50]	2755	7.9 ± 1.1 y (6.0–10.0)	F: 53.7%	Consumption of breakfast, fast foods and savoury snacks (‘never/<once a week’, ‘some days (1–3 days)’, ‘most days (4–6 days)’, ‘every day’)	ΔBMI z-score (IOTF) OV/OB incidence (new cases) and prevalence (total number)	Measurement round, time to follow-up, age at baseline, sex, z-BMI at baseline, abdominal obesity at baseline, school SES, and school urbanisation level	Frequency of eating breakfast and fast foods was not associated with the OV/OB incidence or prevalence or ΔBMIz at FU. Low frequency of eating savoury snacks at BL was associated with decreased OV/OB prevalence at FU (some days vs. every day [OR (95%CI): 0.48 (0.23–0.99)]; never vs. every day [OR (95%CI): 0.27 (0.10–0.72)]); decreased OV/OB risk at FU (some days vs. every day [OR (95%CI): 0.49 (0.24–1.00)]; never vs. every day [OR (95%CI): 0.22 (0.07–0.69)]); decreased ΔBMI z-score (mean change (SD) never vs. every day [β (SD) −0.18 (0.51), p-trend < 0.001])
Berge, 2023, USA [31]	1259	5–9 y	NS	**Quantity:** Consumption of family meals (never, 1–2 times, 3–4 times, 5–6 times, 7 times, >7 times). Family meal interpersonal **quality** (whether during a family meal people (a) watch TV; (b) have conversations; (c) play video games; (d) use tablets/computers; (e) read a book; (f) listen to headphones. A positive interpersonal quality represented conversations without media distraction; a negative interpersonal quality represented no conversations and media distractions	BMI% (CDC)	Household race/ethnicity, parent age, parent gender, and parent educational attainment; models for child and family-level outcomes were additionally adjusted for child age and gender.	Greater weekly family meal quantity at baseline was associated with reduced obesity prevalence at follow-up, with each additional family meal significantly reducing obesity prevalence by 4% [PR (95%CI): 0.96 (0.93–0.99)] Interpersonal quality was not associated with obesity prevalence [0.99 (0.84–1.17)]
Chang and Gable, 2013, USA [32]	6220	11.2 y ± 4.3 mo (10.3–12.8 y)	M: 49% F: 51%	Breakfast at home (times/wk) School-provided foods for lunch (Yes/No) Family dinner (times/wk)	FU BMI (CDC) Weight trajectory groups: (1) stable obese (Obe-Sta); (2) obese to overweight (ObePos1); (3) obese to healthy (ObePos2); (4) stable overweight (OverSta); (5) overweight to healthy (OverPos); (6) overweight to obese (OverNeg); (7) stable healthy (HelSta); (8) healthy to overweight (HelNeg1); and (9) healthy to obese (HelNeg2).	Parental health status, child’s health status, child gender and race, age in months at fifth grade, highest level of parent education in the household, family structure, and household poverty level	Children who ate school-provided lunches less frequently at the 5th grade were more likely to be in ObePos when compared with ObeSta in the 8th grade [OR (95%CI): 1.10 (1.01–1.19)]. Also, children who ate breakfast more frequently at home were more likely to be in OverPos, when compared with OverSta in 8th grade [OR (95%CI): 1.02 (1.00–1.03)]. No other associations between meals and weight trajectories were observed.
Chang and Halgunseth, 2015, USA [33]	6860	11 y	M: 49% F: 51%	Family meal frequency (sum of frequency of breakfast and evening meals) (times/wk) School-provided foods for lunch (“A full meal including salad, soup, a sandwich or a hot meal that is offered each day at a fixed price”: Yes/No)	FU BMI (CDC) Weight status trajectories: (1) stable healthy, (2) stable overweight, (3) healthy change, and (4) unhealthy change.	Parental health status, child’s health status, child gender, ethnicity and acculturation, age in months at fifth grade, and highest level of parent education in the household, family structure, and household poverty level	No association of the frequency of family meals or purchase of school lunches in 5th grade with weight status trajectories in eighth grade.
de la Rie, 2023, Germany, the Netherlands, UK, USA [57]	1275 (Germany) 4007 (the Netherlands) 11,285 (UK) 6740 (USA)	5.2 ± 0.4 y (Germany) 6.1 ± 0.4 y (the Netherlands) 7.2 ± 0.2 y (UK) 7.1 ± 0.4 y (USA)	F: 51.5% (Germany) F: 49.2% (the Netherlands) F: 48.9% (UK) F: 48.6% (USA)	Breakfast consumption (less than 7 d/wk (5 d for US) and 7 d/wk (5 d for US)	ΔBMI (WHO 2007 growth standards)	Parental education, child sex, child age in months (at baseline and follow-up), foreign-born mother, maternal age at the birth of the child, single-parent-household indicator, BMI at baseline, physical activity, screen time	Breakfast consumption significantly predicted BMI in the Netherlands [Regression coefficient (SE): 0.26 (0.14)] and the UK [0.40 (0.13)], but not the USA [0.06 (0.16)], indicating that children in the Netherlands and the UK who ate breakfast daily had lower BMI than children who did not eat breakfast every day. No data for Germany.
Balvin Frantzen, 2013, USA [34]	625	9.1 ± 0.5 y	M: 309 (49%) F: 316 (51%)	Breakfast consumption (Ready-to-eat cereal (RTEC)) (0 = no RTEC breakfast, 1 = 1 d of RTEC breakfast, 2 = 2 d of RTEC breakfast and 3 = 3 days of RTEC breakfast.) *Definition: breakfast was considered the first meal of the morning consisting of any solid food, beverages, or both and named by the respondent as “breakfast”.*	ΔBMI percentile (CDC)	Sex, ethnicity, age, energy, total carbohydrates, and total fat	Frequency of RTEC consumption significantly (*p* = 0.001) affected a child’s BMI (R^2^ change 0.031) with a decrease of 2 percentiles [mean (SD) 1.977 (0.209)] for every day of RTEC consumption. No information regarding other types of breakfast or no breakfast consumption.
Gingras, 2018, USA [23]	995	3.2 y	M: 504 F: 491	Frequency of eating breakfast, eating dinner together with family (“always/daily” versus “≤six times per week”); eating fast food, eating meals while watching television “less than once per week (between zero and three times per month)” versus “≥once per week”.	FU BMI z-score (U.S. national reference data) FU WC FU Whole-BF%, FU trunk fat mass, FU trunk to peripheral fat mass ratio (BIA, DXA)	Mothers’ age, education level, parity, marital status, household income, height and pre-pregnancy BMI (kg/m^2^); child’s sex, race/ethnicity	Eating breakfast daily was associated in both boys and girls with lower BMI-z [β (95%CI)]: [boys −0.13 (−0.24, −0.02). girls −0.13 (−0.23, −0.02)] and DXA BF% [β (95%CI)]: [boys −1.43 (−2.42, −0.45); girls −1.47 (−2.25, −0.68)], and in girls only with lower WC [−1.59 (−2.67, −0.51)], BI BF% [−1.47 (−2.39, −0.54)], DXA trunk fat mass [−0.92 (−1.33, −0.51)] and trunk to peripheral fat ratio [−0.05 (−0.06, −0.03)]. Daily family dinner was associated in girls only with lower BMI-z [β (95%CI)]: [−0.17 (−0.24, −0.11)], WC [−1.14 (−1.80, −0.48)], BI BF% [girls −1.34 (−1.91, −0.77)], DXA trunk fat mass [−0.32 (−0.57, −0.06)] and trunk to peripheral fat ratio [−0.02 (−0.03, −0.01)]. Eating meals while watching television less than once per week was associated in boys with lower BMI-z [β (95%CI)]: [−0.13 (−0.20, −0.05)], WC [−1.55 (−2.39, −0.71)], BI BF% [−1.33 (−1.98, −0.69)], DXA BF% [−1.10 (−1.77, −0.44)], DXA trunk fat mass [−0.56 (−0.88, −0.23)] and trunk to peripheral fat ratio [−0.02 (−0.03, −0.01)]. In girls, eating meals while watching television less than once per week throughout childhood was associated with lower BI BF% [−0.74 (−1.35, −0.14)]. Eating fast foods less than once a week was associated in girls with lower BMI-z [β (95%CI)]: [−0.09 (−0.17, −0.02)], WC [−1.23 (−1.99, −0.48)], DXA BF% [−0.89 (−1.45, −0.33)], DXA trunk fat mass [−0.60 (−0.90, −0.31)] and trunk to peripheral fat ratio [−0.03 (−0.04, −0.02)]
Goetz, 2022, USA [35]	116	4.6 ± 0.9 y	M: 50%	First and last eating events *Definition: First meal in the morning (06:00 to <10:00) and at night (19:00 to <06:00)*	FM, FFM, %BF (DXA)	Age, sex, race and ethnicity, childcare attendance and income-to-needs ratio, BMIz, BL FM, BL %BF	Time of first time eating at baseline was not associated with fat mass at 1 year [effect estimate (95%CI): −0.01 (−0.14, 0.11)] or %BF [−0.1 (−0.50, 0.27)] A later time of last eating event at baseline was associated with increased FM at 1 year [effect estimate (95%CI): 0.17 (0.02, 0.33)] and % BF [0.83 (0.24, 1.42)]
Gopinath, 2016, Australia [55]	699	12.7 y	M: *n* = 319 F: *n* = 380	Frequency of takeaway food (Chinese, fish and chips, hamburger, and chips/fries, pizza) consumption (“less than once per week”, “once per week or more”)	FU BMI, OV/OB categories (IOTF) FU BF% (BIA) FU WC	Ethnicity of the child, country of birth, education, occupation and parental age of both parents, physical activity, screen time	12-year-olds who ate takeaway foods once per week or more compared with those who ate takeaway foods infrequently did not have significantly higher BMI, WC or BF% at 17 years (*p* > 0.05) and did not have significantly higher odds of OV/OB at 17 years, [OR (95%CI): 0.99 (0.59, 1.66) and 1.59 (0.86, 2.94), respectively].
Jaeger, 2022, Belgium, Germany, Italy, Poland, and Spain [53]	729	3 y	F: 53%	Eating occasion (breakfast, lunch, and supper for meals; morning, afternoon, and evening snacks) *Definition: An EO is defined as any occasion where food or beverages are consumed*	ΔBMI z-score (WHO reference guidelines, IOTF)	Parental BMI, country, TEI, misreporting and an interaction term between TEI and country	The redistribution of energy intake with an increase in energy at breakfast, lunch, supper, or snacks as compared to the other EOs was not significantly associated with zBMI [*p* > 0.05].
Juton, 2023, Spain [51]	1400	10.1 ± 0.6 y	M: 692 (49.4%) F: 708 (50.6%)	Meal frequency (3 categories: 5 meals/d, 4 meals/d and <4 meals/d; meals assessed: breakfast, mid-morning snack, lunch, afternoon snack, dinner)	FU BMI z-score FU odds of OV/OB (IOTF) FU WHtR FU odds of AO (WHtR ≥ 0.50)	Sex, age, school, intervention group, maternal education, physical activity, adherence to the Mediterranean diet, baseline zBMI/WHtR	Higher BL meal frequency was associated with lower FU zBMI increase [0.78 (0.61–0.95) for <4 meals/d; 0.67 (0.61–0.73) for 4 meals/d; 0.62 (0.58–0.66) for 5 meals/d] and lower FU WHtR [0.471 (0.467–0.475) for <4 meals/d; 0.465 (0.463–0.468) for 4 meals/d; 0.463 (0.461–0.465) for 5 meals/d]. The FU odds of OV/OB or AO decreased with increase in meal frequency (P for linear trend = 0.035 and 0.028, respectively).
Kelly, 2016, UK [44]	16,936	3 y	F: 8259 (48.8%)	Consumption of sugary drinks (cola, milkshakes, fruit juice) between meals; skipping breakfast	BMI trajectories (stable; decreasing; moderate increasing; high increasing)	Covariates: sociodemographic characteristics	Sugary drink consumption was not a predictor of BMI trajectory. Skipping breakfast in early childhood was associated with higher odds of increasing BMI trajectory [OR (95%CI)]: 1.66 (1.37–2.02) of moderately increasing and 1.76 (1.26–2.56) of highly increasing compared to stable] but also higher odds of decreasing trajectory [OR (95%CI): 2.01 (1.03–3.92)] compared to stable.
Kesztyüs, 2016, Germany [49]	1733 (1212 for the result of interest)	7.1 ± 0.6 y	M: 881 (50.8%) F: 852 (49.2%)	Breakfast frequency before school (never/rarely; often/always)	WHtR	School clustering	Skipping breakfast was not a significant predictor of changes in WHtR [B (SE): 0.36 (0.19)]
Liechty and Lee, 2015, USA [36]	13,568	15.8 ± 1.6 y	M: 6605 (48.7%) F: 6963 (51.3%)	Breakfast skipping (yes/no)	OV/OB onset (change from UW or HW to OV between BL and FU was coded as OV onset. Change from UW, HW or OV to OB was coded as OB onset) [BMI z-score (CDC)]	Age, race/ethnicity, parent education and family structure, BMIz score	Skipping breakfast increased OV onset risk among female adolescents [RR (95%CI): 1.44 (1.00–2.07)] but not male adolescents [RR (95%CI): 0.78 (0.49–1.24)]. Skipping breakfast was not associated with OB onset.
Lipsky, 2015, USA [37]	2785	16.3 ± 0.03 y	M: 45.5% F: 54.5%	Frequency of breakfast consumption (d/wk); family meals (evening) (d/wk); watching TV during meals (d/wk); eating fast foods (d/wk); sweet and salty snacks (times/d)	Prospective 1-year BMI change (next year BMI—current BMI) for waves 1 through 3 Retrospective 1-year BMI change (current BMI—previous year BMI) for waves 2 through 4	Sex, race/ethnicity, Family Affluence Scale (car and computer ownership, family vacations, bedroom sharing), parental educational status, physical activity, time-varying height	There was an inverse association of consumption of sweet and salty snacks with time-varying BMI [βest (SE): −0.33 (0.12), *p* = 0.02]. None of the meal practises (breakfast, family meals, watching TV during meals and fast food) was associated with BMI longitudinally. None of the meal practices were associated with prospective or retrospective 1-year BMI change.
Loren, 2022, USA [38]	8225	Age ‘kindergarden’	M: 51%	Family meal frequency (morning and/or evening meal; d/wk)	FU BMI z-score (CDC)	Race/ethnicity, income-to-needs, sex	Number of family meals per week at BL was not associated with BMI z-score at FU (standardised coefficient γ 0.02, *p* > 0.05)
Mahmood, 2023, Greece, Spain, Bulgaria, Hungary, Belgium, Finland [54]	989	M: 7.3 ± 0.99 y F: 7.4 ± 1.02 y	F: 52%	Frequency of family meals (breakfast, lunch, dinner) (1) three categories (never, 1–2 times/wk, 3–7 times/wk) (2) five categories (never, remained low, decreased, increased, remained high)	ΔBMI = BMI T2 − BMI at BL BMI categories change (normal weight at BL and T2; OV/OB at BL but normal weight at T2, normal weight at BL but OV/OB at T2, OV/OB at BL and T2 [BMI z-score (IOTF)]	Country, group (intervention–control), age and DQ of children, and parental characteristics (age, marital status, educational level, employment, sex, DQ, BMI), family meals frequency and BMI of children at baseline	Increase in family breakfast frequency over time was negatively associated with ΔBMI in girls (β = −0.078, *p* = 0.035) but not boys (β = −0.051, *p* = 0.066). Increase in family dinner frequency was inversely associated with ΔBMI of boys (β = −0.102, *p* = 0.019) and girls (β = −0.198, *p* < 0.001). Boys and girls whose family breakfast frequency increased were more likely to have lower BMI (boys: 0.68; 0.49–0.91; girls: 0.69; 0.34–0.92) than those with a decreased frequency of family breakfasts. A similar association was found between a change in family dinner frequency (boys: 0.57; 0.39–0.83); girls (0.69; 0.42–0.91). The odds of FU OV/OB were decreased for boys [OR (95%CI): 0.76 (0.52, 1.04)] and girls [OR (95%CI)]: 0.72 (0.58, 0.93)] who consumed family breakfasts 3–7 times a week at BL, compared to those who never had breakfasts with family. Having ≥3 family-shared dinners/wk at BL was associated with reduced odds of OV/OB at T2 in boys [OR (95%CI): 0.65 (0.41, 0.96)] and girls [OR (95%CI): 0.53 (0.31–0.87)] compared with those who never shared family dinners during childhood. Increased family breakfast frequency over time was associated with lower odds of OV/OB in boys [OR (95%CI): 0.78 (0.52–1.11)] and girls [OR (95%CI): 0.78 [0.55–1.01]] compared to never having breakfast. Improved family-shared dinners over time showed lower odds of OV/OB at T2 [boys: OR (95%CI): 0.54 (0.33–0.83); girls: 0.61 (0.40–0.97)]. No associations were observed for family lunch meals with any outcome.
Narla and Rehkopf, 2019, USA [39]	2024	10.0 y	F: 2024 (100%)	Exposures assessed at y 1 (BL), 2 and 3 Eating breakfast, morning snack, afternoon snack, evening snack, eating fast food (times/wk) Eating while watching TV, eating with family, eating with homework, eating school lunch, eating alone, skipping lunch, eating with friends, eating in bedroom	BF% (skinfolds)	Participant’s age in months, household income, race, highest level of parental education	Significant adiposity predictors: eating alone (coefficient of association y3: 3.94), skipping lunch (y1: 6.58), (y2: 6.08), (y3: 6.84) Significant protective predictors of adiposity: eating breakfast (coefficient of association y1: −2.14), eating afternoon snack (y1: −4.64), eating evening snack (y1: −2.23), eating while watching TV (y1: −3.41), (y2: −4.24), (y3: −4.03), eating with family (y1: −4.44), (y3: −4.62), eating while doing homework (y2: −5.34), (y3: −5.08), eating with friends (y2: −4.72), (y3: −3.40), eating in bedroom (y2: −3.41) Propensity score matching showed one detrimental adiposity risk factor [skipping lunch at y2: difference score (95%CI): 4.0 (1.1, 6.7); y3: 4.3 (1.6, 7.2)] and five protective factors against adiposity [eating evening snack at y1: −3.1 (−5.9, −0.3), eating with friends at y2: −4.4 (−7.6, −1.4); y3: −6.0 (−10.0, −2.2), eating while watching TV at y2 −5.8 (−10.1, −1.5); y3 −5.3 (−8.5, −2.2), eating while doing homework at y1 −5.7 (−11.1, −0.1); y2 −6.2 (−9.1, −3.5), eating in bedroom at y2 −5.8 (−10.2, −1.2)]
Parkes, 2020, UK [45]	2810	46 mo	M: 1432 F: 1378	Mealtime setting (factor score of three items: main meal eaten in a “dining” area (=kitchen, dining room, combined living/dining room) or “non-dining” area (living room, bedroom, other) (58 and 122 months); mealtime screen use (TV only at 58 months, TV and other screens at 122 months); how often the child sat at a table while eating a main meal (122 months); categories: “formal”, “intermediate” or “informal”, where “informal” indicates greater screen use and less use of dining area.	BMI trajectories: Low Risk, Decreasing Overweight, Increasing Overweight, High/stable Overweight, High/Increasing obesity (IOTF)	Early life factors (child sex, ethnic group, family socioeconomic disadvantage, maternal BMI, child birth order, maternal smoking in pregnancy, maternal mental health, infant feeding), early diet patterns (healthy diet, picky diet), household organisation and routines (home organisation, irregular bedtimes, skipping breakfast), child behaviours at school age (overall screen time, physical activity and sleep)	Informal settings were associated with the FU High/Increasing Obesity and Increasing Overweight trajectories [RRR (95%CI): 3.67 (1.99–6.77); 1.75 (1.17–2.62) respectively]. Intermediate settings were associated with the High/Increasing Obesity and Increasing Overweight trajectories [RRR (95%CI): 1.89 (1.09–3.28); 1.50 (1.03–2.19), respectively].
Stea, 2014, Norway [52]	428	9–10 y (4th grade)	M: 207 F: 221	Meal frequency: (i) CONTINUED skippers (skipping meals at both time points); (ii) START all meals (meals skippers in 4th grade, eat all meals in 7th grade); (iii) STOP all meals (eat all meals in 4th grade, meal skippers in 7th grade); and (iv) ALL meals (eat all meals at both time points). *“All meals” if eating all breakfast/lunch/dinner/evening meals daily; “skipping meals” if eating < 7 d/week*	ΔBMI categories (normal weight and OV) (IOTF)	Maternal education, gender, physical activity, overweight status at 4th grade	Meal skipping was not associated with odds of OV.
Sudharsanan, 2016, USA [40]	6495	NS (5th grade)	M: 50.3%	Eating school breakfast (yes/no)	FU obesity status (CDC) (obesity/non-obesity)	Sex; race/ethnicity, age, physical activity, family socioeconomic status, family marital status, mother’s employment, number of breakfasts and dinners the family ate together in a typical week, school type, urbanicity	Eating breakfast at school was not associated with obesity at FU [OR (95%CI): 1.31; 0.82–1.97]. For children from families below the federal poverty line, eating school breakfast increased the odds of obesity at FU [OR (95%CI): 2.31; 1.25–4.28] compared with children of similar SES who did not receive school breakfast (propensity score matching). A change in school breakfast (from yes to no) between the 5th and 8th grade was not statistically associated with a change in weight status longitudinally.
Taylor, 2017, New Zealand [56]	371	2 y	M: 196 (52.8%) F: 175 (47.2%)	Meal frequency/eating occasion *Definition: a separate eating occasion, the start of the next meal or snack had to be more than 15 min after the end of the previous meal or snack*	BMI z-score (WHO)	Household deprivation and income, maternal parity, mother’s intervention group, infant sex, birth weight, maternal education and pre-pregnancy BMI, smoking during pregnancy, exclusive breast-feeding	Eating frequency at 2 years of age did not predict change in BMI Z-score at FU (difference in BMI Z-score per additional eating occasion 0.02; 95%CI −0.03, 0.06)
Traub, 2018, Germany [48]	1733	7.1 ± 0.6 y	M:881 (50.8%) F:852 (49.2%)	Breakfast consumption before school (“Never and rarely”/“often and always”)	Δweight (kg) ΔBMI percentile (German reference cut-off points) WtHR AO (WHtR ≥ 0.5)	School, migration background, family education level, household income, age, gender, participation in the intervention	Skipping breakfast at BL was positively associated with increases in WHtR [B (SE): 0.50 (0.19)], weight [B (SE): 0.39 (0.12)] and BMI percentile [B (SE): 2.01 (0.90)]. Skipping breakfast was associated with increased odds of FU AO [OR (95%CI): 2.06, 1.23–3.47] and OV [OR (95%CI): 1.71, 1.04–2.80] but not OB [OR (95%CI): 0.90, 0.39–2.07]
Wang, 2017, USA [41]	513	5th grade	M:236 (46%) F: 277 (54%)	Breakfast location patterns [average number of d/wk (0–7) and location where breakfast was eaten] Categories: frequent skippers, inconsistent school eaters, inconsistent home eaters, regular home eaters, regular school eaters, double breakfast eaters	FU BMI percentile (CDC) (Two categories: OV/OB and normal/underweight)	Statistical model: Generalized estimating equation (GEE) models [AOR (95%CI)] Covariates: sex, race/ethnicity, school and study year	Odds of FU OV/OB were higher for students in the skipper group [AOR (95%CI): 2.66 (1.67, 4.24)], inconsistent school eaters [AOR (95%CI): 2.11 (1.29, 3.46)], inconsistent home eaters [AOR (95%CI): 2.02 (1.27, 3.21)] and regular home eaters [AOR (95%CI): 1.70 (1.13, 2.56)] compared with double breakfast eaters.
Wijtzes, 2016, the Netherlands [46]	5913	4 y	M: 2939 (49.7) F: 2974 (50.3)	Meal skipping (breakfast, lunch, dinner; consumption < 7 d/wk). Tracking patterns: stable consumption (consumption at both time points), stable meal skipping (skipping at both time points), decrease in meal skipping (skipping at 4 y and consumption at 6 y); increase in meal skipping (consumption at 4 y and skipping at 6 y). Assessment: self-reported questionnaire (parents)	FU BMI SDS (IOTF) FU FM% (DXA scan)	Child’s sex, age, ethnic background, family socioeconomic position (i.e., maternal educational level, maternal employment status, household income, maternal and paternal BMI, and children’s physical activity, sedentary behaviours and dietary behaviours, BMI at age 4	Breakfast skipping at 4 y was associated with increased FM% at 6 y [β (95%CI): 1.38 (0.36–2.40)]. Continuously measured breakfast skipping at age 4 years was associated with a higher FM% [β (95%CI): 0.64 (0.41–0.88)] and a higher BMI [β (95%CI): 0.08; (0.02–0.13)] at 6 y. Compared with stable breakfast consumers, children in all 3 breakfast-skipping categories had a significantly increased FM% at 6 y [1.80 (0.75–2.85); 1.24 (0.56–1.92); 0.92 (0.11–1.74)] Lunch and dinner skipping at 4 years were not associated with FM at 6 y. No meal skipping at age 4 was associated with BMI SDS at age 6.
Winter, 2016, the Netherlands [47]	Wave 1 (BL) 2230; Wave 2 (T2) 2149; Wave 3 (T3) 1816	11.09 ± 0.56	NS	Breakfast consumption [no regular breakfast (consumed < 5 times/wk; regular breakfast)]	ΔBMI categories (BMI criteria NS)	Gender	Not having regular breakfast at T2 was not associated with OV/OB at T3 [OR (95%CI): 1.41 (0.97–2.06)]

^a^ Presented as mean ± standard deviation or median (1st, 3rd quartile) and/or range, unless otherwise stated. ^b^ Presented as absolute (relative) frequency. Abbreviations: AO: abdominal obesity, BF: body fat, BIA: bioelectrical impedance analysis, BL: baseline, BMI: body mass index, CDC: Centers for Disease Control and Prevention, CI: confidence interval, d: day(s), DQ: diet quality, DXA: dual-energy X-ray absorptiometry, F: female, FFM: fat-free mass, FFQ: food frequency questionnaire, FM: fat mass, FMI: fat mass index, FU: follow-up, HW: healthy weight, IOTF: International Obesity Task Force, M: male, mo: months, NS: not stated, OB: obesity, OR: odds ratio, OV: overweight, PA: physical activity, RR: risk ratio, RRR: relative risk ratio, SDS: standard deviation score, SE: standard error, SES: socioeconomic status, TEI: total energy intake, UK: United Kingdom, USA: United States of America, UW: underweight, WC: waist circumference, WHO: World Health Organisation, WHtR: waist-to-height ratio, wk: week, y: year(s), Δ: change.

**Table 4:** Characteristics of the randomised controlled trial exploring the association between meal patterns and the risk of overweight/obesity.

Study	Population Characteristics			Intervention	Outcomes	Covariates	Results
Author, Year, Country	N	Age ^a^	Sex ^b^				
Polonsky, 2019, USA [42]	793	10.8 ± 1.0 y	M: 662 (48.6%); F: 700 (51.4%)	Design: parallel cluster RCT Groups: IG vs. CG IG (350): breakfast in the classroom, eighteen 45–min nutrition education lessons (importance of breakfast), social marketing materials and corner stores with healthy choices; monthly newsletters to parents (8 schools) CG (443): breakfast offered in the cafeteria before the beginning of the school day and standard education material (8 schools)	OV/OB incidence and prevalence ΔBMI-z (CDC)	Paired stratification of randomisation	There was no difference in combined OV/OB incidence between IG (11.7%) and CG (9.3%) at FU [OR (95%CI): 1.31 (0.85–2.02)]. The OB incidence alone was higher in IG (11.6%) than in CG (4.4%) between BL and FU [OR (95%CI): 2.43 (1.47–4.00)]. There was no difference between IG and CG in the combined OV/OB prevalence or BMI zscore across the study period. The OB prevalence alone at the end point was higher in IG (28.0%) than in CG (21.2%) at FU [OR (95%CI): 1.46 (1.11–1.92)].

^a^ Presented as mean ± standard deviation or median (1st, 3rd quartile) and/or range, unless otherwise stated. ^b^ Presented as absolute (relative) frequency. Abbreviations: BL: baseline, BMI: body mass index, CG: control group, CI: confidence interval, F: female, FU: follow-up, IG: intervention group, M: male, OB: obesity, OV: overweight, RCT: randomised clinical trial, y: year(s), z: z-score, Δ: change.

## Data Availability

Not applicable.

## Supplementary Materials

The following supporting information can be downloaded at https://www.mdpi.com/article/10.3390/children11091100/s1, Supplementary Material S1: PRISMA 2020 checklist; Supplementary Material S2: Detailed search strategy in MEDLINE/Pubmed and Scopus.
