# Supplementary material for: Associations between Meal Patterns and Risk of Overweight/Obesity in Children and Adolescents in Western Countries: A Systematic Review of Longitudinal Studies and Randomised Controlled Trials
Source: Children (Basel). 2024 Sep 7;11(9):1100. doi: 10.3390/children11091100 (PMC11430606; doi:10.3390/children11091100)
Supplement: Supplementary file 1 [file children-11-01100-s001.zip › Meal patterns_OV_MUO risk/Supplementary material 2 - search strategy.docx]

**Search strategy – meal patterns**

**KEYWORDS (title / abstract):**

(“child*” OR “adolescen*” OR “teen*” OR “teenage*” OR “preteen*” OR “youth” OR “pediatric” OR “paediatric” OR “schoolchild*” OR “school age” OR “schoolage*” OR “juvenile*” OR “youngster*” OR “girl” OR “girls” OR “boy” OR “boys” OR “kid” OR “kids” OR “young person*” OR “young people” OR “student*” OR “pupil*”) **AND** (“diet* intervention*” OR “breakfast*” OR “breakfast* composition*” OR “breakfast* frequen*” OR “breakfast* skipping” OR “breakfast* timing*” OR “family breakfast*” OR “dinner*” OR “dinner* frequen*” OR “dinner* composition*” OR “dinner* skipping” OR “dinner* timing*” OR “family dinner*” OR “meal*” OR “meal* pattern*” OR “meal* composition*” OR “meal frequen*” OR “meal* skipping” OR “meal* timing*” OR “meal* habit*” OR “meal* type*” OR “meal* place*” OR “meal* condition*” OR “meal* context*” OR “family meal*” OR “snack*” OR “snack* frequen*” OR “snack* composition*” OR “snack* skipping” OR “snack* timing*” OR “snack* behavio*” OR “family snack*” OR “eating frequen*” OR “eating habit*” OR “home* meal*” OR “eating out” OR “chrononutrition”) **AND** (“weight” OR “body weight” OR “body mass index” OR “BMI” OR “BMI percentile” OR “BMI z-score” OR “overweight” OR “obesity” OR “child* overweight” OR “child* obesity” OR “adipos*” OR “body fat” OR “fatness” OR “skinfold thickness” OR “waist circumference” OR “fat mass” OR “fat free mass” OR “muscle mass” OR “neck circumference” OR “waist-to-height” OR “body composition” OR “weight gain” OR “weight loss” OR “weight status” OR “weight change” OR “metabolically unhealthy obesity” OR “*hypertension” OR “blood pressure” OR “dyslipidemia” OR “dyslipidaemia” OR “cholesterol*” OR “triglyceride*” OR “glycated haemoglobin” OR “glycated haemoglobin” OR “blood glucose” OR “prediabetes” OR “glycemic control” OR “glycaemic control” OR “HOMA” OR “HOMA-IR” OR “insulin resistance” OR “metabolic syndrome” OR “inflammation” OR “liver enzymes” OR “non-alcoholic fatty liver disease” OR “NAFLD” OR “hepatic steatosis” OR “polycystic ovary syndrome” OR “obstructive sleep apnea” OR “obstructive sleep apnoea” OR “uric acid” OR “hyperuricemia” OR “hyperuricaemia”) AND (“prospective*” OR “longitudinal*” OR “randomised clinical trial” OR “randomized clinical trial” OR “randomised controlled trial” OR “randomized controlled trial” OR “relative risk” OR “incidence” OR “risk ratio” OR “RR” OR “hazard ratio” OR “HR”)

**Filters applied in MEDLINE/Pubmed:**

Humans, Child: birth-18 years, Preschool child: 2-5 years, Child: 6-12 years, Adolescent: 13-18 years, English, from 2013/1/1 – 2024/4/30.

**Filters applied in Scopus**

Date: 2013 – present

Language: English

Territory: All European countries, USA, Canada, Australia, New Zealand, undefined

Document type: Article
